# Predicting mTOR Inhibitors with a Classifier Using Recursive Partitioning and Naïve Bayesian Approaches

**DOI:** 10.1371/journal.pone.0095221

**Published:** 2014-05-12

**Authors:** Ling Wang, Lei Chen, Zhihong Liu, Minghao Zheng, Qiong Gu, Jun Xu

**Affiliations:** Research Center for Drug Discovery & Institute of Human Virology, School of Pharmaceutical Sciences, Sun Yat-sen University, Guangzhou, China; University of Granada - Q1818002F, Spain

## Abstract

**Background:**

Mammalian target of rapamycin (mTOR) is a central controller of cell growth, proliferation, metabolism, and angiogenesis. Thus, there is a great deal of interest in developing clinical drugs based on mTOR. In this paper, *in silico* models based on multi-scaffolds were developed to predict mTOR inhibitors or non-inhibitors.

**Methods:**

First 1,264 diverse compounds were collected and categorized as mTOR inhibitors and non-inhibitors. Two methods, recursive partitioning (RP) and naïve Bayesian (NB), were used to build combinatorial classification models of mTOR inhibitors versus non-inhibitors using physicochemical descriptors, fingerprints, and atom center fragments (ACFs).

**Results:**

A total of 253 models were constructed and the overall predictive accuracies of the best models were more than 90% for both the training set of 964 and the external test set of 300 diverse compounds. The scaffold hopping abilities of the best models were successfully evaluated through predicting 37 new recently published mTOR inhibitors. Compared with the best RP and Bayesian models, the classifier based on ACFs and Bayesian shows comparable or slightly better in performance and scaffold hopping abilities. A web server was developed based on the ACFs and Bayesian method (http://rcdd.sysu.edu.cn/mtor/). This web server can be used to predict whether a compound is an mTOR inhibitor or non-inhibitor online.

**Conclusion:**

*In silico* models were constructed to predict mTOR inhibitors using recursive partitioning and naïve Bayesian methods, and a web server (mTOR Predictor) was also developed based on the best model results. Compound prediction or virtual screening can be carried out through our web server. Moreover, the favorable and unfavorable fragments for mTOR inhibitors obtained from Bayesian classifiers will be helpful for lead optimization or the design of new mTOR inhibitors.

## Introduction

Mammalian target of rapamycin (mTOR) is a highly conserved serine/threonine protein kinase (PK) and a vital component of the PI3K/Akt/mTOR signal pathway [1], [2]. mTOR plays a key role in integrating signals from metabolism, energy homeostasis, cell cycle, and stress response. mTOR exists as two complexes, mTORC1 and mTORC2. The mTORC1 complex is composed of Raptor, LST8, PRAS40 and Deptor, and is responsible for the regulation protein synthesis through the phosphorylation of S6K1 and 4E-BP1. The mTORC2 complex consists of Rictor, LST8, SIN1, Deptor and Protor, and regulates cell proliferation and survival through the phosphorylation of Akt/PKB [3], [4].

Rapamycin and its analogues (rapalogues) have successfully been developed as treatments for specific cancers through allosteric binding to the FKBP-12 rapamycin binding (FRB) domain of mTOR. However, recent reports suggest that existing rapalogues do not fully inhibit mTORC1 and do not inhibit mTORC2 [1], [5]. The selective inhibition of mTORC1 by rapalogues has been shown to enhance PI3K signaling through a negative feedback mechanism [6]. This may limit the efficacy of rapalogues. The emerging role of mTORC2 in tumor growth and survival, along with the lack of suppression of this pathway by rapalogues, has led to a great deal of in discovering clinically ATP-competitive mTOR inhibitors that target both mTORC1 and mTORC2, which may offer therapeutic advantages to the rapalogues.

Recently, many potential ATP-competitive inhibitors of mTOR have been discovered [7]–[10]. Based on the selectivity of their inhibition, these compounds are classified into two varieties, namely mTOR-selective inhibitors (dual inhibitors of mTORC1/mTORC2) and dual mTOR/PI3K inhibitors (PI3K is a structurally related enzyme, upstream of mTOR in the signaling pathway). Some mTOR selective inhibitors (e.g., AZD8055 [11], OSI-027 [12], INK-128 [13] and CC-223 [8]) are in clinical trials. PF-04691502 [14], GSK2126458 [15], BEZ235 [16], and XL-765 [17] have begun clinical trials as dual mTOR/PI3K inhibitors. However, marketed ATP-competitive mTOR inhibitors are not available; thus the discovery of novel and diverse scaffolds against mTOR continues to be needed [2], [8], [10].

To date, the assessment of inhibition by anti-mTOR agents (i.e., mTOR inhibitor) on the mTOR signal pathway can be achieved experimentally via *in vitro* or *in vivo* assays [1], [11], [15], [17]. However, these experimental assays are expensive, laborious and time-consuming. They are usually used in later stages of drug design or optimization when the drug candidates exhibit adequate potency and acceptable pharmacokinetic properties. Thus, the development of *in silico* models that provide a rapid and efficient screening platform to identify mTOR inhibitors is vital in the early stages of drug design or optimization.

Some 3D-QSAR and pharmacophore models have been developed to predict ATP-competitive mTOR inhibitors and explain the mechanism of action of some scaffolds. In 2011, Wang and coworkers built a 3D-QSAR based on a morpholinopyrrolopyrimidine scaffold using CoMFA and CoMSIA methods [18]. Their models showed potential predictions that helped in understanding the structure-activity relationship of morpholinopyrrolopyrimidine derivatives and designing new potential mTOR inhibitors based on the morpholinopyrrolopyrimidine scaffold. A similar study was conducted by Karunakar Tanneeru and coworkers based on the triazine scaffold in 2012 [19]. In 2013, Mohammad and coworkers built a series of common features of pharmacophore models based on 6 structurally diverse ATP-competitive mTOR inhibitors. The representative pharmacophore model includes the following four features: a hydrophobic center, an aromatic feature, and four hydrogen bond acceptors [20]. The models exhibit potential to predict inhibitors that are not included in the training set. Similar work was also performed by Karunakar Tanneeru and coworkers, which resulted in four features pharmacophore model (two hydrogen bond acceptors, a hydrophobic center and an aromatic feature) based on 27 ATP-competitive mTOR inhibitors [21]. A disadvantage of 3D-QSAR or SAR models for mTOR inhibitors is the use of a series of compounds based on solely scaffold. Compared with binding modes of ATP-competitive inhibitors based on recently solved crystal structures, these published pharmacophore models are not well consistent with the experimental results [2]. The ATP binding pocket of mTOR is flexible, which makes it difficult to screen new inhibitors based on traditional 3D methods [2], [22], [23]. The broad multi-specificity of mTOR and the lack of an extensive database of ATP-competitive mTOR inhibitors have proven to be almost insurmountable obstacles to establish accurate prediction models. In the present study, we present a large data set of 1,264 molecules that are categorized into ATP-competitive inhibitors and non-inhibitors. *In silico* classification models were constructed using recursive partitioning and naïve Bayesian techniques. The performance and scaffold hopping abilities of *in silico* models were successful validated by external test sets, and these models can be implemented as virtual screening tools in early phases of drug discovery.

## Materials and Methods

### Data Set

The whole date set was collected from the ChEMBL database [24] and BindingDB [25]. The data set was refined with the following criteria: (1) only human mTOR inhibition assay data were selected; (2) only mTOR assay data based on enzyme or enzyme regulation were kept, and allosteric inhibitors were excluded, e.g., rapamycin and its analogs; (3) duplicated compounds and compounds without detailed assay values (K*_i_* or IC_50_) were abandoned. By applying these criteria, a large diverse database containing 1,246 unique compounds was first obtained in our lab. Within this data set, all compounds have K*_i_* or IC_50_ values ranging from 0.08 to 10,000,000 nM. (i.e. nine-order of magnitude). Among these, 1,015 compounds were considered to be “active” in our study as their reported assay values were below 10 µM. Such a cutoff value appeared to be a reasonable starting point for hit-to-lead activity and, in view of the noise level in the data set, the choice of 10 µM would seem justified.

The structures of the compounds were built using MDL ISIS/Draw software. Structures were cross-checked in a search of the Beilstein database and the original published papers. Each molecule in the database was optimized using molecular mechanics (MM) with the MMFF94 force field (Sybyl 7.3). All molecules were saved to the MACCS sdf file and a SMILES database for further analysis. Finally, the whole data set was divided into a training set (964) and test set (300) based on a randomly algorithm in Discovery Studio 3.5 (Accelrys, Inc.). The proportion of training set and test set was about 3 to 1, which was employed in reference [26]. All data are available online: http://rcdd.sysu.edu.cn/mtor/.

### Calculation of molecular descriptors

Herein, thirteen molecular descriptors widely adopted in ADME, QSAR and QSPR predictions were used in our analysis. These descriptors include the octanol/water partitioning coefficient (AlogP) based on Ghose and Crippen's method, the apparent partition coefficient at pH = 7.4 (logD) based on the Csizmadia's method, molecular solubility (logS) based on the multiple linear regression model, molecular weight (MW), the number of hydrogen bond donors (nHBDon), the number of hydrogen bond acceptors (nHBAcc), the number of rotatable bonds (*N_rot_*), the number of rings (nRing), the number of aromatic rings (nAR), the sum of oxygen and nitrogen atoms (N plus O), the molecular polar surface area (MPSA), the molecular fractional polar surface area (MFPSA) and the molecular surface area (MSA). All the descriptors were calculated using the Discovery Studio molecular simulation package (version 3.5, Accelrys Inc., San Diego, CA.).

### Calculation of molecular fingerprints

Here, two types of fingerprints were used to construct the *in silico* model, namely SciTegic extended-connectivity fingerprints (ECFP, FCFP and LCFP) and Daylight-style path-based fingerprints (EPFP, FPFP and LPFP). For each fingerprint class, two diameters 4 and 6, were used in the present study. The smaller diameter 2 was not considered because structural fragments based on a diameter of 2 are small and general. These fingerprints are widely used in other ADME, QSAR and QSPR predictions [26]–[31]. Twelve fingerprints were calculated using the Discovery Studio molecular simulation package (version 3.5, Accelrys Inc., San Diego, CA.).

### Atom center fragments generation

For each compound, the ACFs were derived with the following steps:

1. a heavy atom (non-hydrogen atom) was taken as an atom center for an ACF;
2. atoms *n*-bonds (n≥1) away from the center atom were taken, keeping the bonding topology inside the ACF. If n is 1, it is called as level one ACF (ACF_1_); if n is 2, it is called as level two ACF (ACF_2_); and so on.

Usually, ACF*_n+1_* is larger than ACF*_n_*. Larger ACFs are structurally more specific and result in more accurate prediction, but lose universality. To find a balance point of the accuracy and universality, we generated ACF_1–6_ fragments from the data set using our *in-house* program. ACFs were used as a descriptor that encoded the Bayesian core function to construct a classification model (called ACFs-NB model) based on the *in-house* program. Detailed information of ACFs-NB algorithm is described in *Text S1*. The program can be obtained by request.

### Recursive partitioning

RP is a statistical method for multivariable analysis. It creates a decision tree that strives to correctly classify members of the population based on a dichotomous dependent variable (e.g., inhibition class) and a set of independent variables (e.g., molecular properties and fingerprints). In the present study, 234 RP models were constructed based on 13 molecular properties and 12 fingerprints. 5-fold cross-validation was used to determine the degree of pruning required for the best predictive performance. Detailed descriptions of the RP method can be found in the literatures [26], [32].

### Naïve Bayesian

Bayesian inference derives the posterior probability as a consequence of two antecedents, a prior probability and a “likelihood function” derived from a probability model for the data to be observed. Bayesian inference computes the posterior probability directly based on the core function of eq. 1.
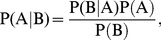
(1)where P(A) is the initial degree of belief in A; P(B) is the initial degree of belief in B; P(A|B) is the degree of belief having accounted for B; and P(B|A) is the degree of belief having accounted for A. Detailed descriptions of the naïve Bayesian method can be found in the literature [33]. In our study, Bayesian analysis and model building were implemented using the Scitegic Pipeline Pilot Laplacian-corrected Bayesian classifier algorithm [28]. This implementation of Bayesian statistics uses information from both the inhibitors (“good”) and non-inhibitors (“bad”) in the training set and removes features from the model that are deemed to be unimportant.

### Performance evaluation of the models

To validate the accuracy and robustness of stability prediction models, a 5-fold cross validation scheme was employed to evaluate the RP, Bayesian and ACFs-NB classifiers. True positives (TP), true negatives (TN), false positives (FP), false negatives (FN), sensitivity (SE), specificity (SP), the prediction accuracy for inhibitors (Q_i_), the prediction accuracy for non-inhibitors (Q_ni_), overall predictive accuracy (Q) and the Matthews correlation coefficient (C) have been calculated. In addition, the receiver operating characteristic (ROC) curve was also plotted. The ROC curve was used to graphically present the model behavior in a visual way. It shows the separation ability of a binary classifier by iteratively setting the possible classifier threshold [34].
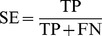
(2)

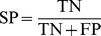
(3)

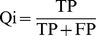
(4)

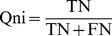
(5)

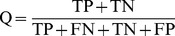
(6)

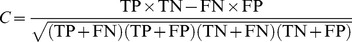
(7)The value of *C* is the most important indicator for the classification accuracy of the models.

## Results and Discussion

### Chemical space and structural diversity analysis

**Figure 1:**
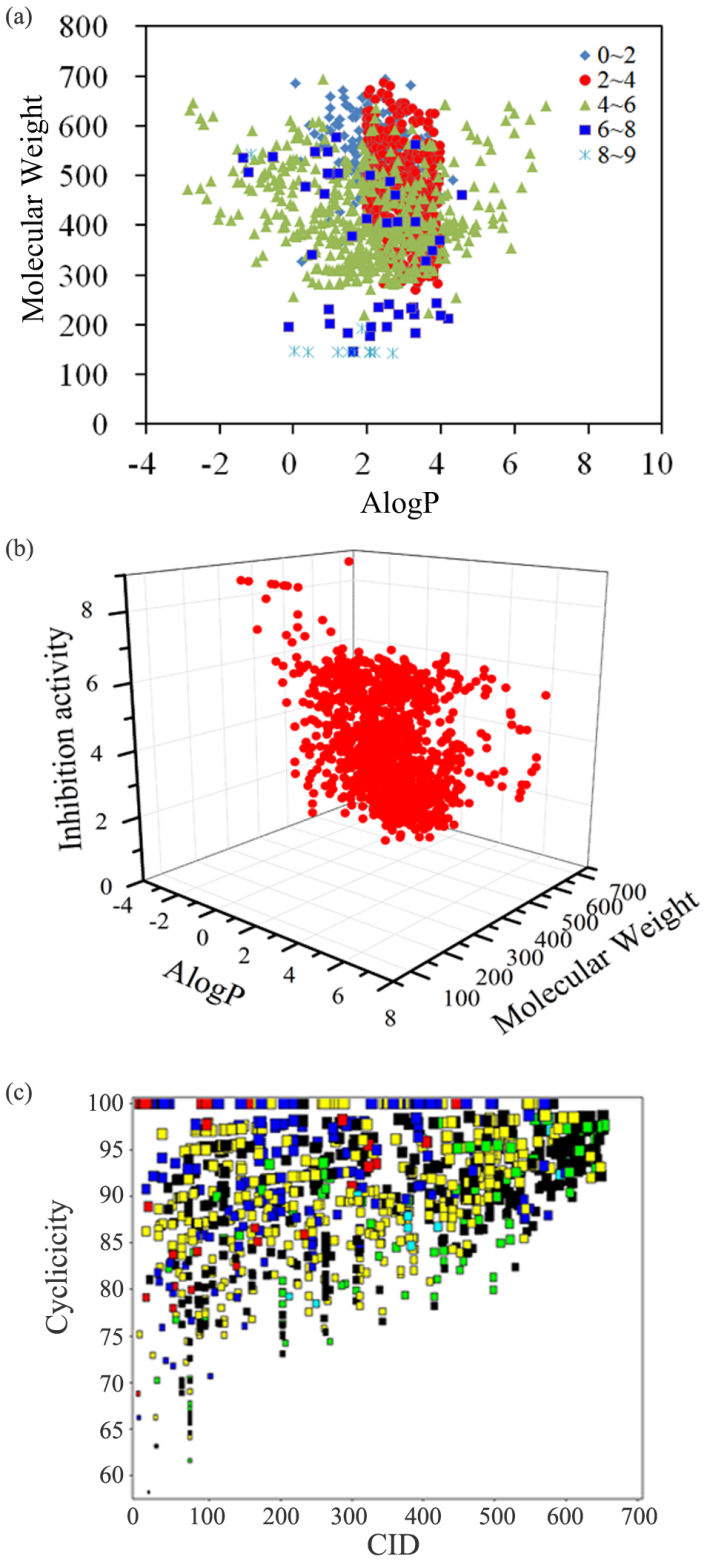
Diversity analysis of the entire data set (n = 1264 compounds) with mTOR inhibitory index. (a) Chemical space defined by molecular weight and AlogP. The data are colored according to the chemical mTOR inhibitory index value. (b) Distribution of mTOR inhibitory index in a chemical space defined by MW and AlogP. Both figures are used the same color scheme. (c) The chemical diversity of 1264 compounds was calculated by *in-house* S-cluster algorithm based on structural features. The color were filled based the number of hydrogen donor of each compound.

The chemical space of the 1,264 compounds is defined in the molecular weight (MW), AlogP, and mTOR inhibitory values of the compounds (Figures 1A and 1B). The structural diversity of the 1,264 compounds was calculated by an *in-house* S-cluster algorithm based on structural features (Figure 1C) [35]. The S-cluster program can be obtained by request. Cyclicity is the metric of the cyclic degree of a compound, where higher cyclicity value means the compound has fewer side chains. Each compound is assigned a compound cluster ID (CID), which is related to the compound's complexity. More complicated compounds have higher CID numbers. Figure 1C suggests that the 1,264 compounds exhibit large chemical structural diversity. The whole data set was randomly split into training set (964) and test set (300).

### Relationships between molecular properties and mTOR inhibition activity

**Figure 2:**
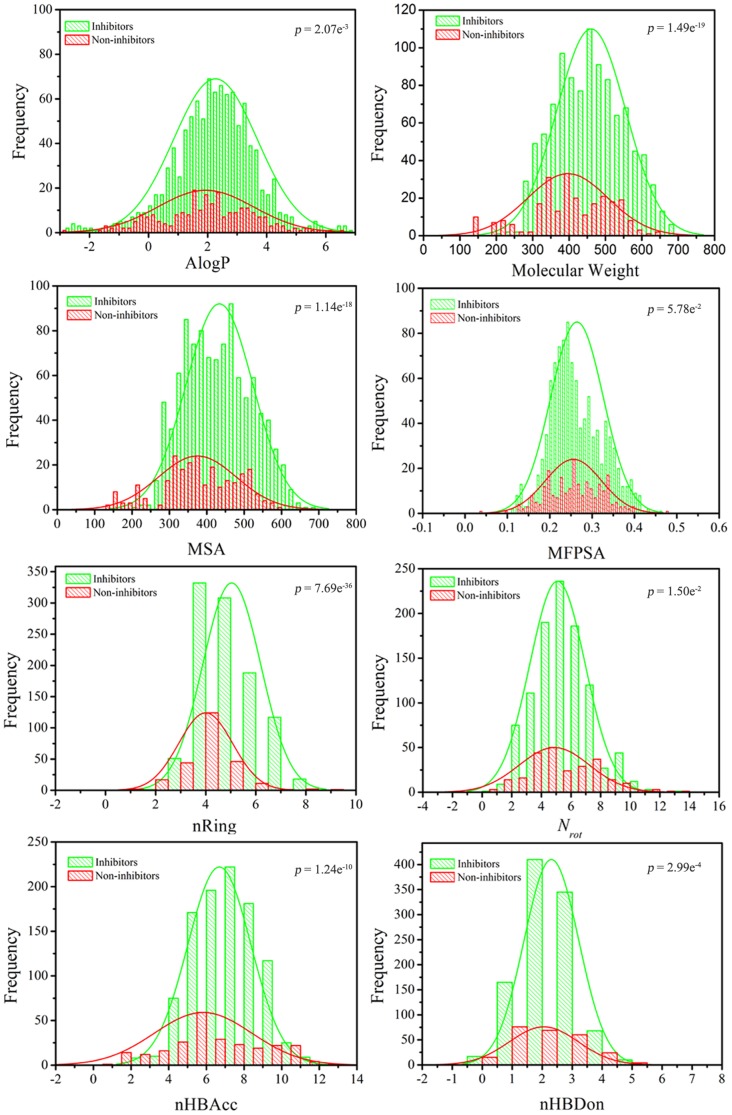
Distributions of eight chemical properties, AlogP, MW, MSA, MFPSA, nRings, *N_rot_*, nHBAcc and nHBDon for mTOR inhibitors and non-inhibitors classes. *p* value: Student's *t* test was used to evaluate the significance of the difference between paired samples and the means.

**Figure 3:**
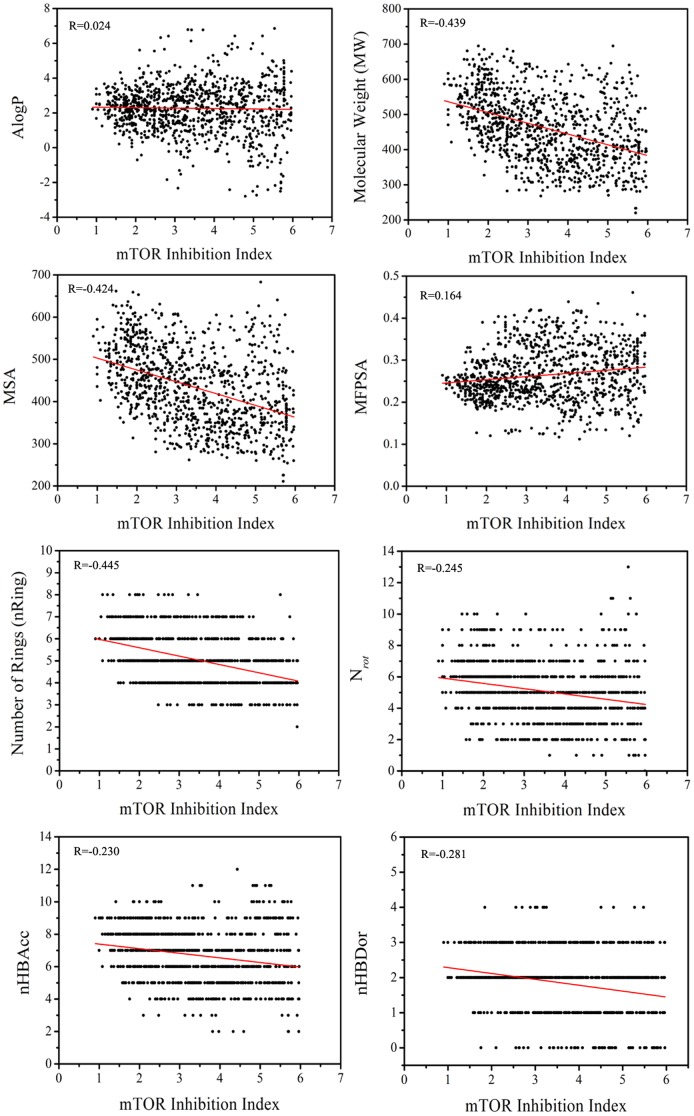
Correlations between eight molecular properties, including AlogP, MW, MSA, MFSA, nRings, *N_rot_*, nHBDor and nHDAcc, and mTOR inhibition index.

A variety of molecular properties, such as lipophilicity, hydrogen bonding ability, molecular flexibility and molecular bulkiness, have been proven to be useful for QSAR, QSPR and ADME predictions [26]–[31]. To increase the interpretability of the models, the relationships between the mTOR inhibition index of 1264 chemicals and 9 key physicochemical descriptors, including ALogP, MW, MSA (molecular surface area), nRing (number of rings), nHBAcc (number of hydrogen bond acceptors), nHBDon (number of hydrogen bond donors), MFPSA (molecular fractional polar surface area), *N_rot_* (the number of rotatable bonds), and N plus O (the sum of oxygen and nitrogen atoms), are presented in Figure 2 and Figure S1. The student's *t* test was used to evaluate the significance of the difference between paired samples and their means. As a complementary test, the linear correlations between each of these nine molecular properties and the mTOR inhibition index (mTOR inhibition index = PIC_50_/PK_i_+2) of 1015 active compounds are shown in Figure 3 and Figure S2.

MW is an estimation of molecular size and complexity. The MW is distributed between 143.18 and 694.83, with a mean of 448.38 (Figure 2). The mean MW values were 460.97 and 396.97 for 1,015 mTOR inhibitors and 249 non-inhibitors, respectively, with a *p* value of 1.49×10^−19^ at the 95% confidence level. These results suggest that MW shows potential classification capability for mTOR inhibitors and non-inhibitors. Similar results are obtained in Figure 3. MW shows a better liner correlation (r = −0.439) with the mTOR inhibition index (1,015 active compounds). As shown in Figure 2, molecules with MW>300 are more likely to be mTOR inhibitors. However, the two MW distributions for inhibitors and non-inhibitors are still strongly overlapped. The MSA of chemicals indicated a highly significant difference of the mean MSA of mTOR inhibitors and non-inhibitors as shown by the *p* value of 1.14×10^−18^ (Figure 2). The mean values of MSA were 375.16 and 433.98 for non-inhibitors and inhibitors, respectively, and it has a good linear correlation with the mTOR inhibition index (r = −0.424). This result indicates that molecules with low MSA are unfavorable for mTOR inhibition (e.g., MSA<250).

nRings can be considered as a descriptor that characterizes the complexity or bulkiness of a molecule, because a larger molecule usually has more rings. As shown in Figure 2, the nRings of the chemicals suggests a significant difference between the mean nRings of mTOR inhibitors and non-inhibitors with a *p* value of 7.69×10^−36^. In fact, nRings of the molecules has a relatively obvious linear correlation with the mTOR inhibition index (r = −0.445). Similar results can be obtained based on the analysis of N plus O (Figure S1 and Figure S2). Hydrogen binding ability is commonly represented by nHBAcc and nHBDon. The *p* values for the mean nHBAcc and nHBDon values for mTOR inhibitors and non-inhibitors were 1.24×10^−10^ and 2.99×10^−4^, respectively, indicative of minor significant difference for nHBDon. Compared to the nHBDon contribution, nHBAcc plays an important role in the classification of mTOR inhibitors and non-inhibitors. Our findings are well consistent with the recently X-ray experimental results (only nHBAcc was observed in three classes mTOR inhibitors) [2]. Based on the *p*-value and linear correlations (Figure 2 and 3), the other three descriptors (AlogP, MFPSA and *N_rot_*) do not show any capability to discriminate between mTOR inhibitors and non-inhibitors.

Based on the analysis above, it is obvious that using individual or several simple chemical descriptors is not good criteria for classifying mTOR inhibitors and non-inhibitors.

### Performance of recursive partitioning models

**Figure 4:**
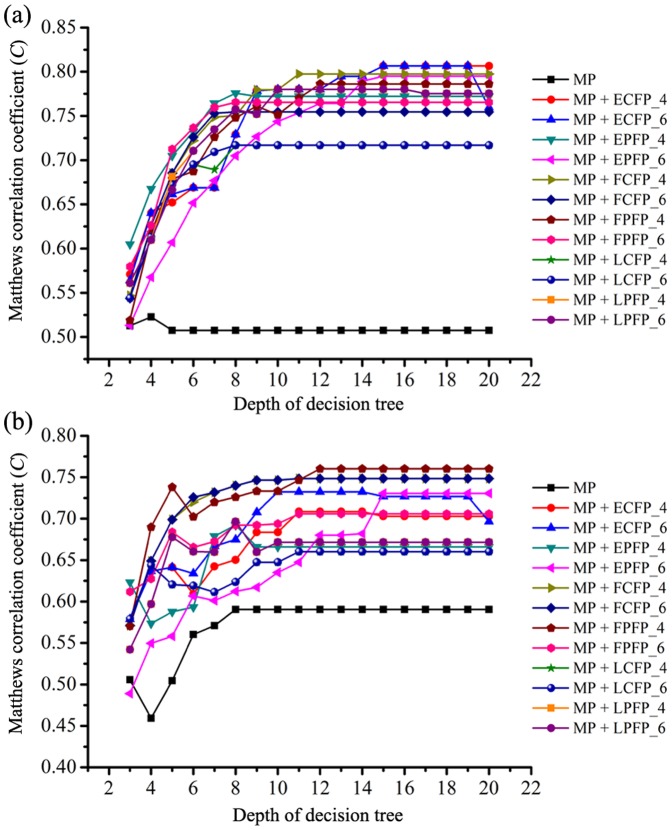
The Matthews correlation coefficient (*C*) versus the tree depth for (a) the training set and (b) the test set. The 243 RP models were constructed based on molecular properties (thirteen molecular descriptors) and the different combinational of molecular properties and twelve different fingerprint sets.

To develop more precise and understandable classification models, the RP technique was used to establish decision trees to classify mTOR inhibitors and non-inhibitors. Compared with “the blind operations” of the ANN and SVM methods, the RP results can be converted into simple hierarchical rule trees that are easily understood. In RP analysis, the depth of the decision tree is a key parameter that dominates its complexity. Usually, larger tree depth can increase the accuracy on the training data but risks over-fitting, while small depth tends to increase the applicability of a tree to new data sets, but at the risk of decreased accuracy and failing to identify important features in the training data [26]. The best tree depth parameter should be defined according to the predictions for the test data. In present study, the tree depth was changed from 3 to 20 and the corresponding performance of 234 RP models on training and test sets was evaluated (Figure 4). The 5-fold cross-validation technique was used to evaluate the model robustness.

First, 18 decision tree models were constructed based on the thirteen molecular properties (MP). According to the Matthews correlation coefficient (*C*) value from the test set, the best tree depth is 8 (Figure 4 and Table S2). The performance of the best RP model based on MP is shown in Table 1. For the training set, the sensitivity and specificity are 82.0% and 76.5%, the *C* value and AUC are 0.505 and 0.846, and the prediction accuracy of the model in terms of correspondence to the test set (SE_test_ = 82.4%, SP_test_ = 85.5%, *C* = 0.590 and AUC = 0.851, Table 1) is comparable with that of training set. However, the low *C* and AUC values suggest that the best RP models based on MP may not have good well prediction accuracy for inhibitors or non-inhibitors (50.5% for training set and 55.8% for test set, Table 1). Molecular properties (MP) can depict whole-molecule properties, but they cannot characterize the important substructures or molecular fragments that play a key role in mTOR inhibition. Therefore, a combination of MP and molecular fingerprints were used simultaneously to establish RP models. Here, 216 RP models based different combinations of 12 sets of fingerprints with MP were constructed and evaluated (Figure 4, Table S1 and Table S2). Obviously, the addition of fingerprints can improve the performance of the RP models because the *C* values of the RP models based on fingerprints and MP are higher than those of RP models solely based on MP (Figure 4 and Table S1). For different combinations fingerprints and MP, the performances of the best RP models were screened according to the *C* value from different tree depth (Figure 4 and ). For different fingerprint, the best tree depth parameters are different. The performance of the 12 best RP models based on 12 fingerprints and MP are summarized in Table 1. According to the *C* values listed in Table 1 for 300 tested compounds, the fingerprint set FPFP_4 performs better than the others, indicated by the highest *C* value (0.760). The best tree depth parameter is 12. The best RP model based on FPFP_4 and MP has a sensitivity of 90.8%, a specificity of 91.9%, and a prediction accuracy of 97.7% for the mTOR inhibitor class, and a prediction accuracy of 72.2% for the non-inhibitor class, and an overall prediction accuracy of 91.0%. The performance evaluation of the model on the training set also show comparable results with that of test set (Table 1). Moreover, the AUC values were 0.982 and 0.937 for the training set and test set, respectively.

**Table 1:** Performance of the best RP classification models for the training set and test set using different combinational of molecular properties and fingerprints.

Descriptors	Training set										Test set									
	TP	FN	TN	FP	SE	SP	Q_i_	Q_ni_	C	AUC	TP	FN	TN	FP	SE	SP	Q_i_	Q_ni_	C	AUC
MP^a^	637	140	143	44	0.820	0.765	0.935	0.505	0.508	0.846	196	42	53	9	0.824	0.855	0.956	0.558	0.590	0.851
MP+ECFP_4	709	68	177	10	0.912	0.947	0.986	0.722	0.780	0.946	210	28	56	6	0.882	0.903	0.972	0.667	0.708	0.898
MP+ECFP_6	709	68	177	10	0.912	0.947	0.986	0.722	0.780	0.946	210	28	58	4	0.882	0.935	0.981	0.674	0.732	0.917
MP+EPFP_4	707	70	177	10	0.910	0.947	0.986	0.717	0.776	0.980	213	25	53	9	0.895	0.855	0.959	0.679	0.692	0.941
MP+EPFP_6	714	63	178	9	0.919	0.952	0.988	0.739	0.795	0.974	220	18	52	10	0.924	0.839	0.957	0.743	0.731	0.873
MP+FCFP_4	715	62	178	9	0.920	0.952	0.988	0.742	0.797	0.959	216	22	56	6	0.908	0.903	0.973	0.718	0.748	0.917
MP+FCFP_6	699	78	176	11	0.900	0.941	0.985	0.693	0.755	0.945	216	22	56	6	0.908	0.903	0.973	0.718	0.748	0.929
MP+FPFP_4	710	67	178	9	0.914	0.952	0.987	0.727	0.786	0.982	216	22	57	5	0.908	0.919	0.977	0.722	0.760	0.937
MP+FPFP_6	704	73	176	11	0.906	0.941	0.985	0.707	0.765	0.944	215	23	53	9	0.903	0.855	0.960	0.697	0.706	0.891
MP+LCFP_4	692	85	170	17	0.891	0.909	0.976	0.667	0.717	0.942	210	28	52	10	0.882	0.839	0.955	0.650	0.660	0.895
MP+LCFP_6	692	85	170	17	0.891	0.909	0.976	0.667	0.717	0.942	210	28	52	10	0.882	0.839	0.955	0.650	0.660	0.995
MP+LPFP_4	709	68	177	10	0.912	0.947	0.986	0.722	0.780	0.966	206	32	55	7	0.866	0.887	0.967	0.632	0.672	0.903
MP+LPFP_6	709	68	171	16	0.912	0.914	0.978	0.715	0.757	0.943	210	28	55	7	0.882	0.887	0.968	0.663	0.696	0.895

a MP represents thirteen molecular properties. The best tree depth is 8 for RP models (MP, MP+EPFP_4 and MP+LPFP_6), 10 for RP models (MP+ECFP_6 and MP+LPFP_4), 11 for RP models (MP+FCFP_4, MP+FCFP_6, MP+LCFP_4, MP+LCFP_6 and MP+FPFP_6) and 12 for RP model (MP+FPFP_4). The detailed results can be found in Table S1 and Table S2 in Supporting Information.

**Figure 5:**
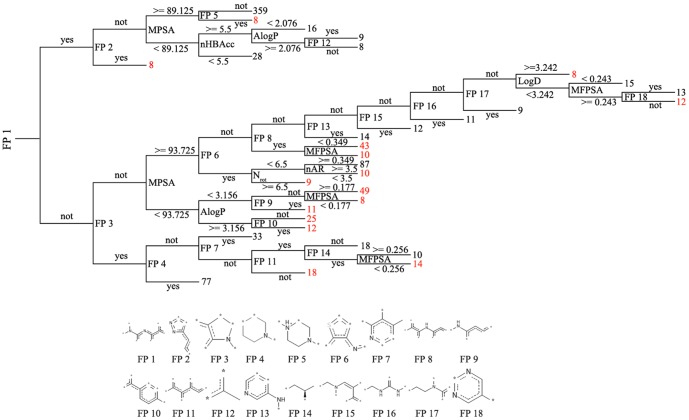
Decision tree to classify compounds into mTOR inhibitor and non-inhibitor classes based on best RP method. The decision tree was constructed using combinational MP and FPFP_4 fingerprint, and the tree depth is 12. FP: Fingerprint; yes: contain this fingerprint; not: not contain; red font represents non-inhibitors; black font represents inhibitors.

The best decision tree, with a tree depth of 12 based on FPFP_4 and MP, is shown in Figure 5. The discriminant descriptors include seven MPs and 18 structure fragments. Of the seven MPs chosen by the decision tree, AlogP and logD are properties that describe molecule hydrophobicity, *N_rot_* and nAR describe the molecule's bulkiness, and MFPSA, MPSA and nHBAcc describe its electrostatic properties or hydrogen binding ability. In other words, the molecular hydrophobicity, size and electrostatic properties are important for mTOR inhibition, which is consistent with previously 3D-QSAR results [18], [19]. Moreover, the eighteen fragments based on FPFP_4 fingerprint also play a key role in discriminating between mTOR inhibitors and non-inhibitors (Figure 5).

### Performance of naïve Bayesian classifier models

The naïve Bayesian classifier is an unsupervised learner that does not have a fitting process and tuning parameters, unlike RP method that is sensitive to predefined parameters, e.g., tree depth. The process of Bayesian learning is to search through each feature in an unbiased way for those with separation power.

**Figure 6:**
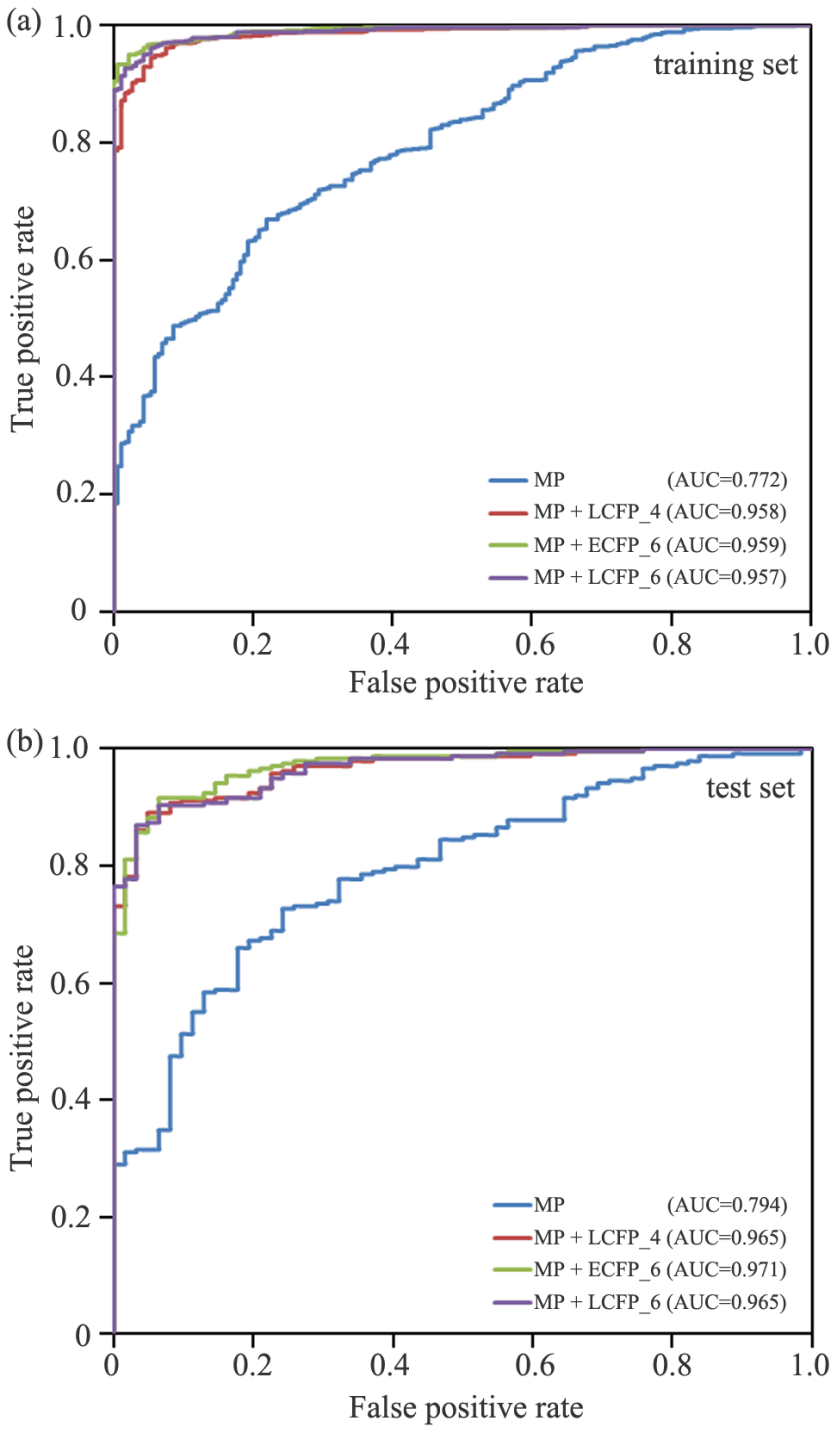
The performance of different models depicted graphically via receiver operating characteristic (ROC) plot of Bayesian model based on molecular properties (MP) and fingerprints for (a) training set and (b) for test set.

Similar to the RP analysis, the performance of the naïve Bayesian classifier based on MP and fingerprints was evaluated. Detailed results are summarized in Table 2. According to the *C* values determined by the leave-one-out (LOO) cross-validation, the performance of the Bayesian models based on 12 fingerprints and MP is quite different for the training set (*C*:0.422∼0.832). MP+ECFP_4, MP+ECFP_6 and MP+LCFP_6 are associated with better classifiers. The best classifier based MP and ECFP_6 fingerprint set has a sensitivity of 94.1%, a specificity of 94.7%, a prediction accuracy for mTOR inhibitors of 98.7% and a prediction accuracy for mTOR non-inhibitors of 79.4% for the training set. Compared with the Bayesian classifier based solely on MP, the addition of fingerprints can significantly improve the classification (Figure 6). All the Bayesian models were validated by the performance of the external 300 tested compounds, and the detailed results are listed in Table 2. Three models (MP+ECFP_6, MP+FPFP_6 and MP+LCFP_6) are good classifiers. Compared with the prediction accuracy of the RP models, the best Bayesian classifier performs slightly better. For the 300 tested compounds, the best Bayesian classifier based on MP and LCFP_6 fingerprint set retrieves a sensitivity of 90.3%, a specificity of 93.6%, and an overall prediction accuracy of 91.0%. The best Bayesian classifier has a slightly better *C* and AUC values (0.765 and 0.965) for the test set compared to that of the best RP classifier (*C* = 0.760, AUC = 0.937). Similar results can be found for the training set (Table 1 and Table 2).

**Table 2:** Performance of the 13 Bayesian classification models for the training set and test set using different combinational of molecular properties and fingerprints.

Descriptors	Training set										Test set									
	TP	FN	TN	FP	SE	SP	Q_i_	Q_ni_	C	AUC	TP	FN	TN	FP	SE	SP	Q_i_	Q_ni_	C	AUC
MP^a^	673	140	121	66	0.828	0.647	0.911	0.464	0.422	0.772	163	75	48	14	0.685	0.774	0.921	0.390	0.378	0.794
MP+ECFP_4	730	47	174	13	0.940	0.930	0.983	0.787	0.818	0.958	205	33	59	3	0.861	0.952	0.986	0.641	0.714	0.970
MP+ECFP_6	731	46	177	10	0.941	0.947	0.987	0.794	0.832	0.959	214	24	58	4	0.899	0.935	0.982	0.707	0.758	0.971
MP+EPFP_4	713	64	157	30	0.918	0.840	0.960	0.710	0.712	0.921	185	53	54	8	0.777	0.871	0.959	0.505	0.548	0.916
MP+EPFP_6	662	115	179	8	0.852	0.957	0.988	0.609	0.695	0.932	190	48	56	6	0.798	0.903	0.969	0.538	0.597	0.945
MP+FCFP_4	699	78	174	13	0.900	0.930	0.982	0.690	0.747	0.955	206	32	57	5	0.866	0.919	0.976	0.640	0.696	0.96
MP+FCFP_6	722	55	176	11	0.929	0.941	0.985	0.762	0.806	0.958	209	29	57	5	0.878	0.919	0.977	0.663	0.714	0.967
MP+FPFP_4	698	79	164	23	0.898	0.877	0.968	0.675	0.706	0.926	187	51	57	5	0.786	0.919	0.974	0.528	0.595	0.934
MP+FPFP_6	687	90	168	19	0.884	0.898	0.973	0.651	0.699	0.932	188	50	57	5	0.790	0.919	0.974	0.533	0.600	0.952
MP+LCFP_4	721	56	176	11	0.928	0.941	0.985	0.759	0.804	0.958	209	29	59	3	0.878	0.952	0.986	0.670	0.738	0.965
MP+LCFP_6	718	59	180	7	0.924	0.963	0.990	0.753	0.812	0.957	215	23	58	4	0.903	0.935	0.982	0.716	0.765	0.965
MP+LPFP_4	661	116	180	7	0.851	0.963	0.990	0.608	0.697	0.948	204	34	59	3	0.857	0.952	0.986	0.634	0.708	0.964
MP+LPFP_6	695	82	180	7	0.894	0.963	0.990	0.687	0.762	0.949	206	32	58	4	0.866	0.935	0.981	0.644	0.708	0.969

a MP represents thirteen molecular properties.

**Figure 7:**
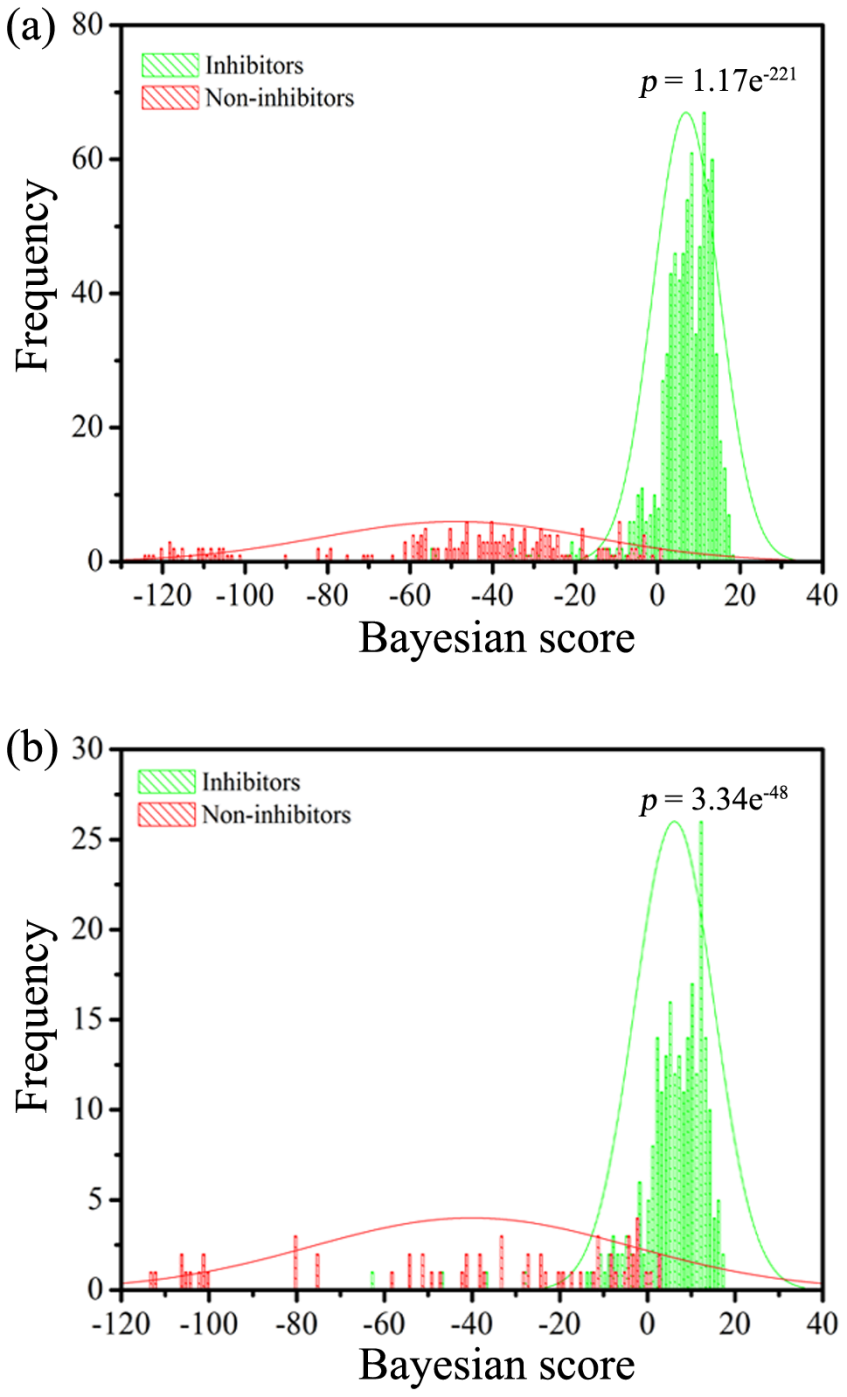
The distributions of Bayesian score predicted by the Bayesian classifier based on molecular properties and the LCFP_6 fingerprint set for the inhibitor and non-inhibitor classes for (a) the training set and (b) the test set. The Bayesian scores for the training set were obtained by sing the leave-one-out (LOO) cross-validation process.

The Bayesian score based on MP and LCFP_6 was used to evaluate the discrimination of inhibitors from non-inhibitors via bimodal histograms of the training and test data sets (Figure 7). As shown in Figure 7a, the *p* value associated with the difference in the mean Bayesian score of training set mTOR inhibitors versus non-inhibitors was 1.17×10^−221^ at the 95% confidence level, suggesting that the two distributions are significantly different. The Bayesian score of inhibitor tends to have more positive value, while the Bayesian score of non-inhibitor tends to have more negative value. Similar results can be found in the 300 tested compounds (Figure 7b). For virtual screening, the Bayesian score can be a quantitation standard to select new potential mTOR inhibitors (like docking, pharmacophore or shape-feature score). For both the training and test sets, the Bayesian score of both classes of compounds have some overlaps between −20 and 0. This region can be defined as the “uncertain zone”, indicating that when the Bayesian score of a compound is located in this region, the prediction for this compound may be not reliable. In other words, a Bayesian score is greater than zero that can be used as a cutoff value to select new mTOR inhibitors for a virtual screening project.

### Performance of ACFs-NB models

Recently, virtual screening tools were developed in our lab based on atom center fragments (ACFs) approach [36]–[40]. A program (called ACFs_NB), which can classify compounds into actives and non-actives based on ACFs and Bayesian rules, has been implemented in our lab. Here, we constructed a classifier that discriminates between mTOR inhibitors and non-inhibitors using ACFs_NB program. The 5-fold cross-validation technique was used to evaluate the model's robustness. The detailed results of the ACFs method are shown in Table S3. The different *C* values were obtained based on different ACFs layers. The best ACFs model has a sensitivity of 92.4%, a specificity of 90.3%, a mTOR inhibitor predictivity of 97.3%, a mTOR non-inhibitor predicativity of 75.7%, and an overall predictivity of 92.0% for the test set when the ACF-layer is set to 3. Compared the best RP and naïve Bayesian classifiers (Table 1 and Table 2), ACFs showed a good prediction abilities because it has a slightly better *C* and AUC values (0.777 and 0.968, Table S3). A web-based service for predicting mTOR inhibitors or non-inhibitors was developed based on the ACFs method (called mTORPredictor) and can be accessed at http://rcdd.sysu.edu.cn/mtor/.

### Privileged fragments for mTOR inhibition activity

**Figure 8:**
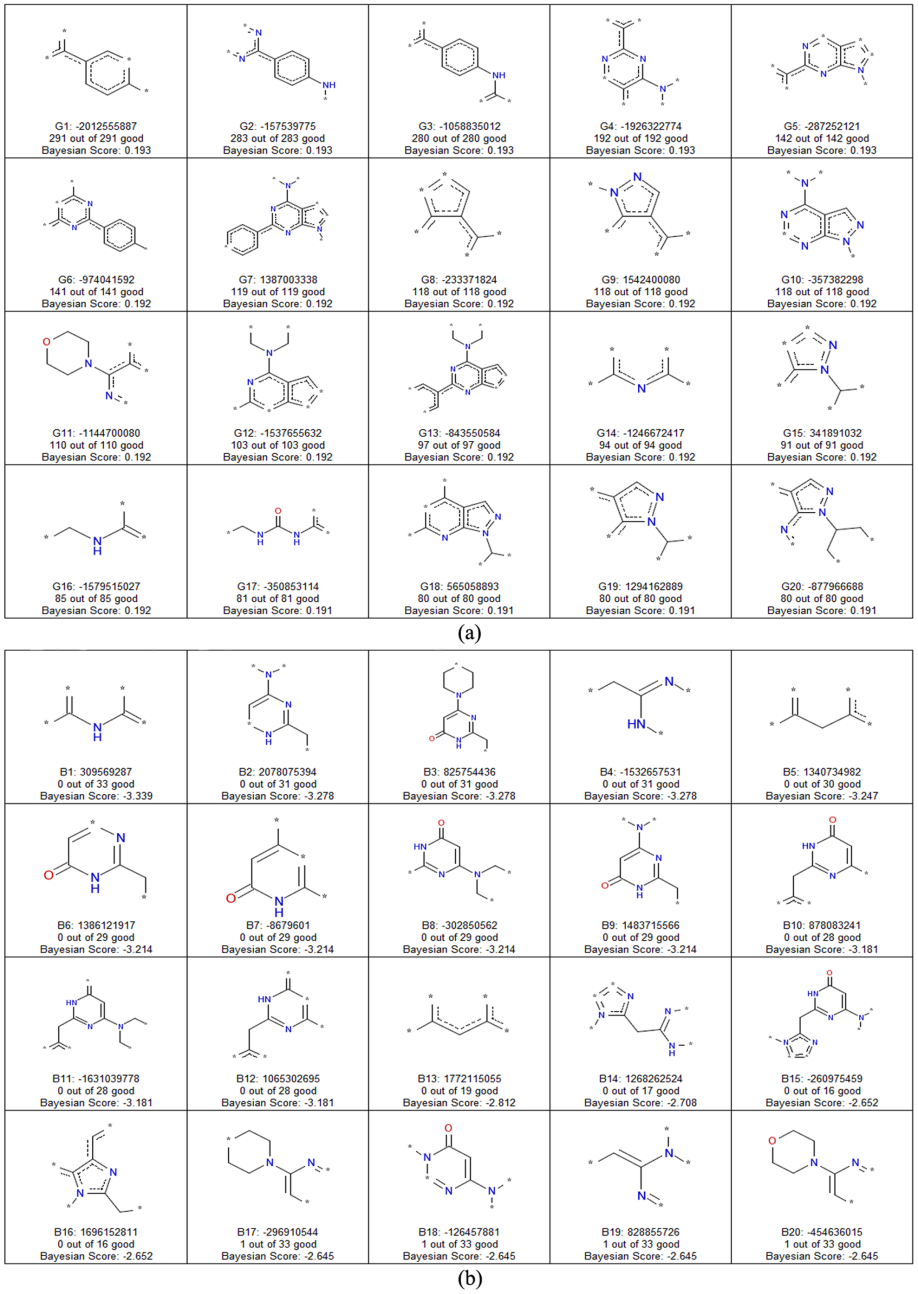
Important favorable and unfavorable fragments for mTOR inhibition obtained from Bayesian classifiers. (a) Selected 20 fragments with incremental effect, prefixed with “G”, on mTOR inhibition (b) selected 20 fragments with detrimental effect, prefixed with “B”, on mTOR inhibition, predicted by the best Bayesian model based on molecular properties and LCFP_6 fingerprints set. The frequency of their occurrences in active (good) molecules is given in bracket, with * represents any atom.

To further explore favorable or unfavorable structural fragments for mTOR inhibition, the fingerprints were translated into 2D fragments. The privileged fragments given by the best Bayesian classifier (MP and LCFP_6) may be useful for medicinal chemists when designing molecules with better mTOR inhibition. The top 20 favorable and 20 unfavorable fragments ranked by their Bayesian scores are summarized in Figure 8.

Analysis of the fragments with positive contributions to mTOR inhibition in Figure 8a showed that many fragments have nitrogen atoms encoded in saturated rings or connected with saturated rings (except fragments 11, 16, and 17). Obviously, the nitrogen atoms in these key fragments can serve as strong hydrogen acceptors and form stable H-bonding interactions with the mTOR kinase domain. Furthermore, these fragments may be as “support scaffolds” that assist in maintaining the active conformation and form favorable hydrophobic interactions with mTOR. Our findings are consistent with the recent published co-crystallized complex of mTOR kinase and inhibitors (PDB ID: 4JSX and 4JT5) [2]. The oxygen atom in fragment 11 is an electron donor and therefore acts as an H-bond acceptor, which is also validated by experimental X-ray results (PDB ID: 4JT6). Fragment 17 contains urea or carbamate groups, indicating that these fragments may act as H-bond acceptors or donors to form H-bond interactions with mTOR. Arie Zask et. al. observed a similar result based on SAR analysis, homolog modeling, and molecular docking technique [41].

The 20 fragments shown in Figure 8b indicate that the existence of these fragments is unfavorable for mTOR inhibition. It is quite interesting that many fragments have nitrogen atoms encoded in unsaturated rings or connected with unsaturated rings. These unsaturated rings encoded in unfavorable fragments may not be as “support scaffolds” because of its flexibility. Another reason is that the proton of nitrogen atoms in unsaturated rings is not necessary for mTOR inhibition, which is consistent with our nHBDon analysis, two published pharmacophore models [20], [21] and recent X-ray results [2]. Moreover, 8 unfavorable fragments contain a lactam group of unsaturated rings, indicating that the lactam group may play a key role in unfavorable mTOR inhibition. Fragments 14 and 16 are fragments that have nitrogen atoms in five saturated rings, but they are cataloged in unfavorable class. The major reason may be due to the nitrogen atoms connection environment (ortho-connection for favorable and meta-connection for unfavorable) or substituents are not from active scaffold. Our results may be useful for designing molecules with better mTOR inhibition.

### Scaffold hopping and experimental validation of classification models

The generalization ability of a model determines its usefulness and reliability. In the present study, the performances of the RP, Bayesian, and ACFs-NB models were validated by an external 300 tested compounds with 5-fold cross-validation. To further prove our models are reliable and useful, we predicted 37 compounds with mTOR inhibition activity published recently (all compounds show IC_50_<10 µM) [42]–[44]. In a blind test, 37 new inhibitors (Table 3) were predicted using the top three best RP, Bayesian models, and the best ACFs model. The detailed results are summarized in Table S3. As shown in Table S3, RP models (MP+ECFP_6, MP+FCFP_4, and MP+FPFP_4) achieved ∼97.3% accuracy rate, while Bayesian (MP+ECFP_6, MP+LCFP_4, and MP+LCFP_6) and ACFs-NB models had a 100% accuracy rate. These results demonstrate that our models are reliable and useful.

**Table 3:** The predictions for the 37 tested compounds using the top three RP, and Bayesian and ACFs-NB classifiers^a^.

Cmps	Expt.	RP_ECFP_6	RP_FCFP_4	RP_FPFP_4	NB_ECFP_6	NB_LCFP_4	NB_LCFP_6	ACFs-NB
1	1	1	1	1	1	1	1	1
2	1	1	1	1	1	1	1	1
3	1	1	1	1	1	1	1	1
4	1	1	1	1	1	1	1	1
5	1	1	1	1	1	1	1	1
6	1	1	1	1	1	1	1	1
7	1	1	1	1	1	1	1	1
8	1	1	1	1	1	1	1	
9	1	1	1	1	1	1	1	1
10	1	1	1	1	1	1	1	1
11	1	1	1	1	1	1	1	1
12	1	1	1	1	1	1	1	1
13	1	1	1	1	1	1	1	1
14	1	1	1	1	1	1	1	1
15	1	1	1	1	1	1	1	1
16	1	1	1	1	1	1	1	
17	1	1	1	1	1	1	1	1
18	1	1	1	1	1	1	1	1
19	1	1	1	1	1	1	1	1
20	1	1	1	1	1	1	1	1
21	1	1	1	1	1	1	1	1
22	1	1	1	1	1	1	1	1
23	1	1	1	1	1	1	1	1
24	1	1	1	1	1	1	1	1
25	1	1	1	1	1	1	1	1
26	1	1	1	1	1	1	1	1
27	1	1	1	1	1	1	1	1
28	1	1	1	1	1	1	1	1
29	1	1	1	1	1	1	1	1
30	1	1	0	0	1	1	1	1
31	1	1	1	1	1	1	1	1
32	1	1	1	1	1	1	1	1
33	1	1	1	1	1	1	1	1
34	1	0	1	1	1	1	1	1
35	1	1	1	1	1	1	1	1
36	1	1	1	1	1	1	1	1
37	1	1	1	1	1	1	1	1

a 0 represents mTOR noninhibitor and 1 represents inhibitor; Expt: experimental results.

**Figure 9:**
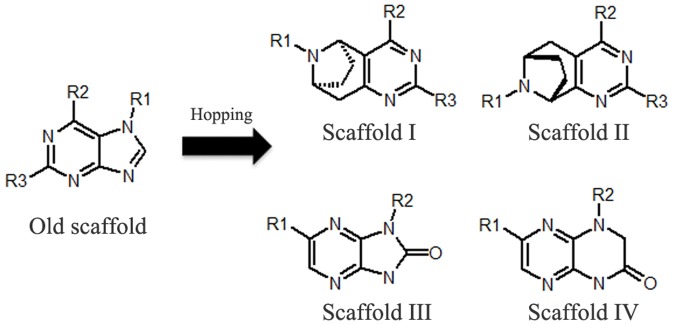
New scaffolds (right) were hopped based on old scaffold (left, contained in train set and test set). R1, R2 and R3 were substituents.

Among the 37 compounds, 18 were novel inhibitors from a hit-to-lead discovery strategy [42]. Nineteen compounds were derived from structural modification of old scaffolds [43], [44]. Four novel scaffolds and one old scaffold are listed in Figure 9. Scaffolds I and II have similar substituents to the old scaffold (R2 and R3 group, Figure 9 and Table S3). Scaffolds III and IV differ not only in substituents (R1 and R2) but also in the position of substituents. Eighteen new inhibitors based on four novel scaffolds were all predicted correctly, indicating that scaffold hopping can be carried out via virtual screening our models.

### Active cutoff value effects

In the present study, compounds were considered to be “inhibitors” if their reported IC_50_ or K_i_ were below 10 µM; this cutoff value appeared to be a reasonable starting point for hit-to-lead activity. To estimate the influence of these active cutoff values on the performance of the classification models, two other threshold values (1 and 5 µM) were used to split the data into inhibitor and non-inhibitor classes. The classification models based on best RP (MP+FPFP_4) and Bayesian (MP+LCFP_6) parameters were reconstructed, and detailed results are listed in Table S4. As shown in Table S4, for RP models, the best model was established based on the 10 µM cutoff value according to the *C* value from the training set (*C* = 0.762 for 1 µM, 0.772 for 5 µM, and 0.786 for 10 µM). Similar results were found for the 300 tested compounds. For the Bayesian models, the best model was constructed based on a 1 µM cutoff value for the training set (*C* = 0.759 for 1 µM, 0.734 for 5 µM, and 0.753 for 10 µM), while similar results are obtained from the test set (Table S4). The tree Bayesian models show an overall prediction accuracy of 89.3%, 87.6%, and 91.0% for the 1 µM, 5 µM, and 10 µM models, respectively. It should be noted that the cutoff values are arbitrarily defined, and we cannot determine which values are best. Similar results were found for the ACFs-NB model (Table S3, three cutoff values). However, as shown in Table S3 and Table S4, classification models with reliable predictive ability for the tested compounds can be obtained even when a different threshold value was used. Based on the above analysis, we selected active cutoff value of 10 µM not only because this value appeared to be a reasonable starting point for hit-to-lead activity but also because it represents the active level of compounds from the current virtual screening project.

### Application of the RP, Bayesian, and ACFs-NB models

Based on important information from the RP, Bayesian, and ACFs-NB models, there are at least three applications of RP and Bayesian models. In the simplest sense, the favorable fragments presented in Figure 8a can be used as queries for screening compound libraries. Furthermore, the results of the models could be useful for the design and optimization of compounds with mTOR inhibition activity by replacing unfavorable fragments with favorable fragments, removing inactive fragments altogether, or adding active fragments to other fragments with promising mTOR inhibitory activity. In addition, the best RP, Bayesian, and ACFs-NB models are well-suited as tools to predict whether a compound is an mTOR inhibitor and for virtual screening. Compound prediction or virtual screening can be carried out through our web server (http://rcdd.sysu.edu.cn/mtor/).

### Conclusions

In the present study, we report an extensive ATP-competitive mTOR inhibition database consisting of 1,264 molecules. On the basis of the diversity set of mTOR inhibition data, the relationships between thirteen important molecular properties and mTOR inhibition have been systematically examined. We observed that some of the properties, especially molecular weight, MSA, nRings, and a sum of N plus O atoms, are important contributors to mTOR inhibition, but no single molecular property is sufficient to distinguish inhibitors from non-inhibitors. The RP technique was applied to construct the decision trees to classify the whole data set into inhibitor and non-inhibitor classes. To characterize the structural features important for mTOR inhibition, structural fingerprints were introduced into our analysis. We found that the introduction of fingerprints significantly improves the prediction accuracy. Then, Bayesian categorization modeling was applied to establish classifiers for mTOR inhibition. The best Bayesian classifier based on MP and LCFP_6 fingerprint achieved high prediction accuracies for the training set and the test set (overall prediction accuracy of 93.2% for 964 compounds in the training set using a leave-one-out cross-validation procedure and 91.0% for the 300 compounds in the test set). Finally, an ACFs-NB classifier was constructed based on an in-house algorithm, achieving overall prediction accuracy of 92.0% for 300 tested compounds. The scaffold hopping abilities of the best RP, Bayesian, and ACFs-NB models were successfully evaluated via predicting 37 recently published new mTOR inhibitors. Comparing the performance and scaffold hopping abilities of the best RP and Bayesian models, the ACFs-NB classifier is comparable or slightly better than the RP and Bayesian methods. Therefore, a web server for predicting mTOR inhibitors or non-inhibitors was developed based on the ACFs and NB method. The important favorable or unfavorable fragments for mTOR inhibition provided by the Bayesian classifiers will be very helpful in lead optimization or the design of new inhibitors with better mTOR inhibitory activity.

## Supporting Information

Figure S1 **Distributions the sum of N plus O atom counts for mTOR inhibitors and non-inhibitors. Student's** ***t*** **test was used to evaluate the significance of the difference between paired samples and the means.**
(TIF)

Figure S2 **Correlations between the sum of N plus O atom counts and mTOR inhibition index.**
(TIF)

Figure S3 **Structures of the predictions for the 37 tested compounds using the top three RP, and Bayesian and ACFs-NB classifiers.**
(TIF)

Table S1 **The classification performance of RP models for training set based on the matthews correlation coefficient (** ***C*** **) using different tree depth.**
(DOC)

Table S2 **The classification performance of thirteen RP models for test set based on the matthews correlation coefficient (** ***C*** **) using different tree depth.**
(DOC)

Table S3 **The classification performance of ACFs classifiers for test set.**
(DOC)

Table S4 **The classification performance of RP and Bayesian classifiers based on three cutoff values.**
(DOC)

Text S1 **Detailed information of of ACFs-NB algorithm.**
(DOC)
