# Supplementary figures and images for: Predicting mTOR Inhibitors with a Classifier Using Recursive Partitioning and Naïve Bayesian Approaches
Source: PLoS One. 2014 May 12;9(5):e95221. doi: 10.1371/journal.pone.0095221 (PMC4018356; doi:10.1371/journal.pone.0095221)

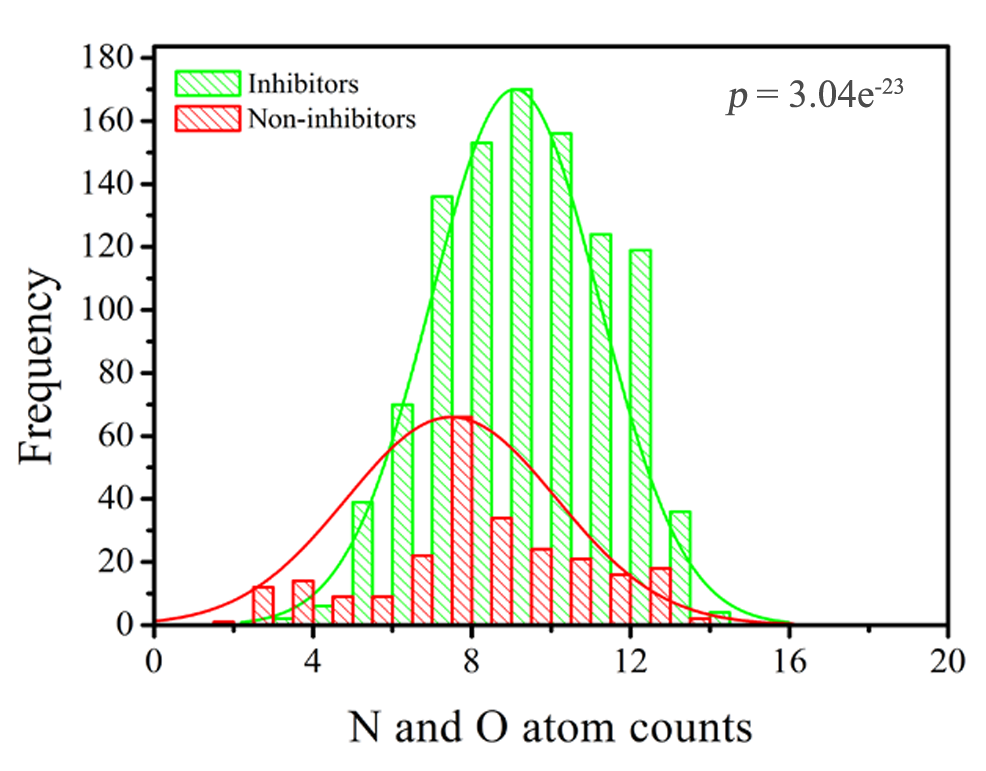

Supplement: Figure S1 — Distributions the sum of N plus O atom counts for mTOR inhibitors and non-inhibitors. Student's t test was used to evaluate the significance of the difference between paired samples and the means. (TIF) [file pone.0095221.s001.tif]

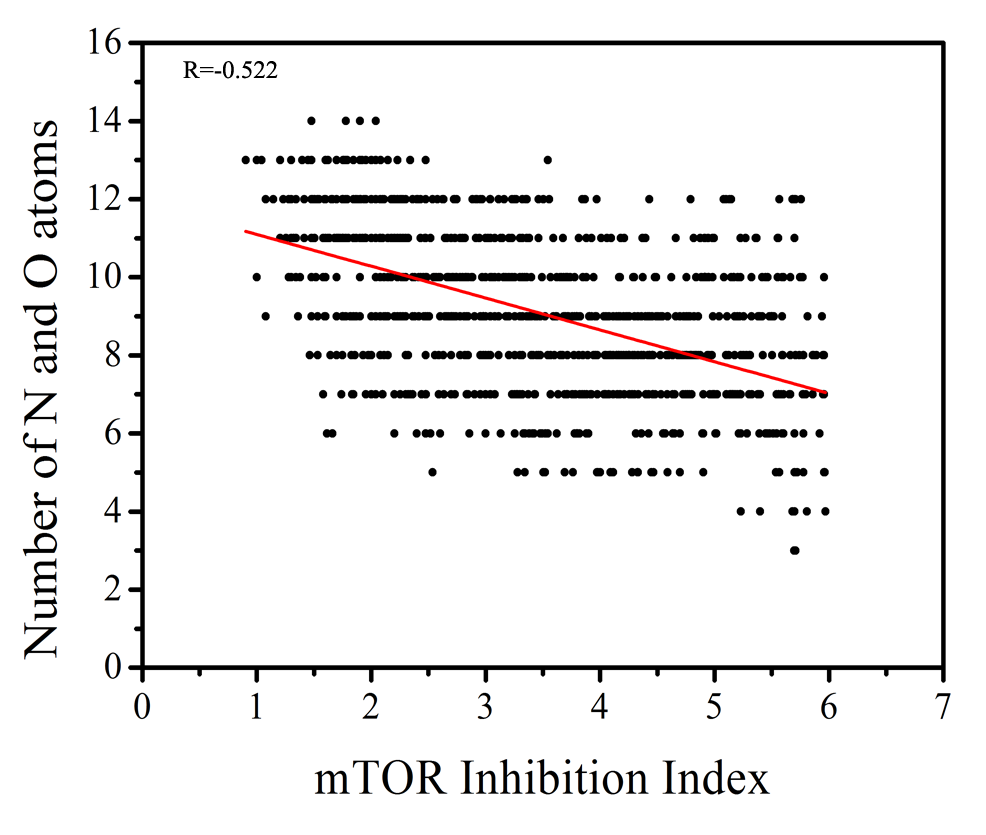

Supplement: Figure S2 — Correlations between the sum of N plus O atom counts and mTOR inhibition index. (TIF) [file pone.0095221.s002.tif]
