# Supplementary material for: Predicting mTOR Inhibitors with a Classifier Using Recursive Partitioning and Naïve Bayesian Approaches
Source: PLoS One. 2014 May 12;9(5):e95221. doi: 10.1371/journal.pone.0095221 (PMC4018356; doi:10.1371/journal.pone.0095221)
Supplement: Table S1 — The classification performance of RP models for training set based on the matthews correlation coefficient ( C ) using different tree depth. (DOC) [file pone.0095221.s004.doc]

**Table S1.** The classification performance of RP models for training set based on the matthews correlation coefficient (*C*) using different tree depth.

| Depth | MP*^a^* | MP/ECFP_4 | MP/ECFP_6 | MP/EPFP_4 | MP/EPFP_6 | MP/FCFP_4 | MP/FCFP_6 | MP/FPFP_4 | MP/FPFP_6 | MP/LCFP_4 | MP/LCFP_6 | MP/LPFP_4 | MP/LPFP_6 |
| --- | --- | --- | --- | --- | --- | --- | --- | --- | --- | --- | --- | --- | --- |
| 3 | 0.513 | 0.571 | 0.564 | 0.605 | 0.513 | 0.547 | 0.544 | 0.519 | 0.580 | 0.544 | 0.544 | 0.561 | 0.561 |
| 4 | 0.523 | 0.640 | 0.640 | 0.667 | 0.568 | 0.626 | 0.626 | 0.618 | 0.626 | 0.613 | 0.613 | 0.610 | 0.610 |
| 5 | 0.508 | 0.652 | 0.661 | 0.705 | 0.607 | 0.685 | 0.685 | 0.679 | 0.713 | 0.672 | 0.672 | 0.681 | 0.666 |
| 6 | 0.508 | 0.669 | 0.669 | 0.732 | 0.652 | 0.722 | 0.726 | 0.687 | 0.737 | 0.695 | 0.695 | 0.711 | 0.711 |
| 7 | 0.508 | 0.669 | 0.669 | 0.764 | 0.677 | 0.749 | 0.753 | 0.726 | 0.760 | 0.689 | 0.709 | 0.735 | 0.735 |
| 8 | 0.508 | 0.729 | 0.729 | 0.776 | 0.705 | 0.751 | 0.755 | 0.748 | 0.765 | 0.717 | 0.717 | 0.757 | 0.757 |
| 9 | 0.508 | 0.779 | 0.779 | 0.772 | 0.727 | 0.780 | 0.755 | 0.761 | 0.765 | 0.717 | 0.717 | 0.752 | 0.752 |
| 10 | 0.508 | 0.780 | 0.780 | 0.772 | 0.744 | 0.780 | 0.755 | 0.751 | 0.765 | 0.717 | 0.717 | 0.780 | 0.780 |
| 11 | 0.508 | 0.780 | 0.780 | 0.772 | 0.754 | 0.797 | 0.755 | 0.770 | 0.765 | 0.717 | 0.717 | 0.780 | 0.780 |
| 12 | 0.508 | 0.780 | 0.780 | 0.772 | 0.764 | 0.797 | 0.755 | 0.786 | 0.765 | 0.717 | 0.717 | 0.780 | 0.780 |
| 13 | 0.508 | 0.795 | 0.795 | 0.772 | 0.764 | 0.797 | 0.755 | 0.786 | 0.765 | 0.717 | 0.717 | 0.780 | 0.780 |
| 14 | 0.508 | 0.795 | 0.795 | 0.772 | 0.789 | 0.797 | 0.755 | 0.786 | 0.765 | 0.717 | 0.717 | 0.780 | 0.780 |
| 15 | 0.508 | 0.807 | 0.807 | 0.772 | 0.795 | 0.797 | 0.755 | 0.786 | 0.765 | 0.717 | 0.717 | 0.780 | 0.780 |
| 16 | 0.508 | 0.807 | 0.807 | 0.772 | 0.795 | 0.797 | 0.755 | 0.786 | 0.765 | 0.717 | 0.717 | 0.780 | 0.780 |
| 17 | 0.508 | 0.807 | 0.807 | 0.772 | 0.795 | 0.797 | 0.755 | 0.786 | 0.765 | 0.717 | 0.717 | 0.775 | 0.775 |
| 18 | 0.508 | 0.807 | 0.807 | 0.772 | 0.795 | 0.797 | 0.755 | 0.786 | 0.765 | 0.717 | 0.717 | 0.775 | 0.775 |
| 19 | 0.508 | 0.807 | 0.807 | 0.772 | 0.795 | 0.797 | 0.755 | 0.786 | 0.765 | 0.717 | 0.717 | 0.775 | 0.775 |
| 20 | 0.508 | 0.807 | 0.760 | 0.772 | 0.795 | 0.797 | 0.755 | 0.786 | 0.765 | 0.717 | 0.717 | 0.775 | 0.775 |

*^a^*MP : molecular properties.
