# Supplementary material for: Predicting mTOR Inhibitors with a Classifier Using Recursive Partitioning and Naïve Bayesian Approaches
Source: PLoS One. 2014 May 12;9(5):e95221. doi: 10.1371/journal.pone.0095221 (PMC4018356; doi:10.1371/journal.pone.0095221)
Supplement: Table S2 — The classification performance of thirteen RP models for test set based on the matthews correlation coefficient ( C ) using different tree depth. (DOC) [file pone.0095221.s005.doc]

**Table S2.** The classification performance of thirteen RP models for test set based on the matthews correlation coefficient (*C*) using different tree depth.

| Depth | MP*^a^* | MP/ECFP_4 | MP/ECFP_6 | MP/EPFP_4 | MP/EPFP_6 | MP/FCFP_4 | MP/FCFP_6 | MP/FPFP_4 | MP/FPFP_6 | MP/LCFP_4 | MP/LCFP_6 | MP/LPFP_4 | MP/LPFP_6 |
| --- | --- | --- | --- | --- | --- | --- | --- | --- | --- | --- | --- | --- | --- |
| 3 | 0.506 | 0.579 | 0.579 | 0.623 | 0.489 | 0.571 | 0.571 | 0.571 | 0.612 | 0.579 | 0.579 | 0.542 | 0.542 |
| 4 | 0.459 | 0.637 | 0.637 | 0.574 | 0.550 | 0.649 | 0.649 | 0.690 | 0.627 | 0.644 | 0.644 | 0.597 | 0.597 |
| 5 | 0.504 | 0.641 | 0.641 | 0.588 | 0.558 | 0.699 | 0.699 | 0.738 | 0.684 | 0.620 | 0.620 | 0.678 | 0.678 |
| 6 | 0.560 | 0.609 | 0.634 | 0.593 | 0.607 | 0.720 | 0.726 | 0.702 | 0.666 | 0.619 | 0.619 | 0.660 | 0.660 |
| 7 | 0.571 | 0.642 | 0.667 | 0.679 | 0.601 | 0.732 | 0.732 | 0.720 | 0.672 | 0.611 | 0.611 | 0.660 | 0.660 |
| 8 | 0.590 | 0.650 | 0.675 | 0.692 | 0.612 | 0.740 | 0.740 | 0.726 | 0.692 | 0.624 | 0.624 | 0.696 | 0.696 |
| 9 | 0.590 | 0.684 | 0.708 | 0.666 | 0.617 | 0.747 | 0.747 | 0.733 | 0.692 | 0.647 | 0.647 | 0.660 | 0.660 |
| 10 | 0.590 | 0.684 | 0.732 | 0.666 | 0.635 | 0.747 | 0.747 | 0.733 | 0.694 | 0.647 | 0.647 | 0.672 | 0.672 |
| 11 | 0.590 | 0.708 | 0.732 | 0.666 | 0.647 | 0.748 | 0.748 | 0.747 | 0.706 | 0.660 | 0.660 | 0.672 | 0.672 |
| 12 | 0.590 | 0.708 | 0.732 | 0.666 | 0.680 | 0.748 | 0.748 | 0.760 | 0.706 | 0.660 | 0.660 | 0.672 | 0.672 |
| 13 | 0.590 | 0.708 | 0.732 | 0.666 | 0.680 | 0.748 | 0.748 | 0.760 | 0.706 | 0.660 | 0.660 | 0.672 | 0.672 |
| 14 | 0.590 | 0.708 | 0.732 | 0.666 | 0.682 | 0.748 | 0.748 | 0.760 | 0.706 | 0.660 | 0.660 | 0.672 | 0.672 |
| 15 | 0.590 | 0.703 | 0.727 | 0.666 | 0.731 | 0.748 | 0.748 | 0.760 | 0.706 | 0.660 | 0.660 | 0.672 | 0.672 |
| 16 | 0.590 | 0.703 | 0.727 | 0.666 | 0.731 | 0.748 | 0.748 | 0.760 | 0.706 | 0.660 | 0.660 | 0.672 | 0.672 |
| 17 | 0.590 | 0.703 | 0.727 | 0.666 | 0.731 | 0.748 | 0.748 | 0.760 | 0.706 | 0.660 | 0.660 | 0.672 | 0.672 |
| 18 | 0.590 | 0.703 | 0.727 | 0.666 | 0.731 | 0.748 | 0.748 | 0.760 | 0.706 | 0.660 | 0.660 | 0.672 | 0.672 |
| 19 | 0.590 | 0.703 | 0.727 | 0.666 | 0.731 | 0.748 | 0.748 | 0.760 | 0.706 | 0.660 | 0.660 | 0.672 | 0.672 |
| 20 | 0.590 | 0.703 | 0.697 | 0.666 | 0.731 | 0.748 | 0.748 | 0.760 | 0.706 | 0.660 | 0.660 | 0.672 | 0.672 |

*^a^*MP : molecular properties.
