# Supplementary material for: Predicting mTOR Inhibitors with a Classifier Using Recursive Partitioning and Naïve Bayesian Approaches
Source: PLoS One. 2014 May 12;9(5):e95221. doi: 10.1371/journal.pone.0095221 (PMC4018356; doi:10.1371/journal.pone.0095221)
Supplement: Table S3 — The classification performance of ACFs classifiers for test set. (DOC) [file pone.0095221.s006.doc]

**Table S3.** The classification performance of ACFs classifiers for 300 tested compounds.

| Layer | TP | FN | TN | FP | SE | SP | Qi | Qni | *C* | Q | AUC |
| --- | --- | --- | --- | --- | --- | --- | --- | --- | --- | --- | --- |
| 1 | 216 | 22 | 37 | 25 | 0.908 | 0.597 | 0.896 | 0.627 | 0.514 | 0.843 | 0.907 |
| 2 | 221 | 17 | 53 | 9 | 0.929 | 0.855 | 0.961 | 0.757 | 0.750 | 0.913 | 0.962 |
| 3*^a^* | 220 | 18 | 56 | 6 | 0.924 | 0.903 | 0.973 | 0.757 | 0.777 | 0.920 | 0.968 |
| 4 | 219 | 19 | 56 | 6 | 0.920 | 0.903 | 0.973 | 0.747 | 0.770 | 0.917 | 0.958 |
| 5 | 218 | 20 | 57 | 5 | 0.916 | 0.919 | 0.978 | 0.740 | 0.774 | 0.917 | 0.957 |
| 6 | 215 | 23 | 56 | 6 | 0.903 | 0.903 | 0.973 | 0.709 | 0.742 | 0.903 | 0.956 |
| 3*^b^* | 181 | 12 | 88 | 19 | 0.938 | 0.822 | 0.905 | 0.880 | 0.773 | 0.897 | 0.945 |
| 3*^c^* | 205 | 19 | 67 | 9 | 0.915 | 0.882 | 0.958 | 0.779 | 0.766 | 0.907 | 0.949 |

*^a^*The compound was categorized into inhibitor when mTOR inhibition value is less than 10 µM; *^b^*The compound was categorized into inhibitor when mTOR inhibition value is less than 1µM; *^c^*The compound was categorized into the inhibitor when mTOR inhibition value is less than 5 µM.
