# Supplementary material for: Predicting mTOR Inhibitors with a Classifier Using Recursive Partitioning and Naïve Bayesian Approaches
Source: PLoS One. 2014 May 12;9(5):e95221. doi: 10.1371/journal.pone.0095221 (PMC4018356; doi:10.1371/journal.pone.0095221)
Supplement: Table S4 — The classification performance of RP and Bayesian classifiers based on three cutoff values. (DOC) [file pone.0095221.s007.doc]

**Table S4.** The classification performance of RP and Bayesian classifiers based on three active cutoff values.

| Descriptors | Training set | | | | | | | | | |  | Test set | | | | | | | | | |  |
| --- | --- | --- | --- | --- | --- | --- | --- | --- | --- | --- | --- | --- | --- | --- | --- | --- | --- | --- | --- | --- | --- | --- |
|  | TP | FN | TN | FP | SE | SP | Q_i_ | Q_ni_ | C | AUC |  | TP | FN | TN | FP | SE | SP | Q_i_ | Q_ni_ | C | AUC | |
| Model 1*^a^* | 565 | 79 | 291 | 29 | 0.877 | 0.910 | 0.951 | 0.7865 | 0.762 | 0.952 |  | 170 | 23 | 83 | 24 | 0.881 | 0.776 | 0.876 | 0.783 | 0.658 | 0.859 | |
| Model 2*^b^* | 650 | 75 | 222 | 17 | 0.897 | 0.929 | 0.975 | 0.7475 | 0.772 | 0.958 |  | 202 | 22 | 58 | 18 | 0.902 | 0.763 | 0.918 | 0.725 | 0.654 | 0.892 | |
| Model 3*^c^* | 710 | 67 | 178 | 9 | 0.914 | 0.952 | 0.988 | 0.7265 | 0.786 | 0.982 |  | 216 | 22 | 57 | 5 | 0.908 | 0.919 | 0.977 | 0.722 | 0.760 | 0.937 | |
| Model 4*^d^* | 549 | 95 | 300 | 20 | 0.852 | 0.938 | 0.965 | 0.759 | 0.756 | 0.931 |  | 173 | 20 | 95 | 12 | 0.896 | 0.888 | 0.935 | 0.826 | 0.773 | 0.961 | |
| Model 5*^e^* | 641 | 84 | 232 | 7 | 0.884 | 0.971 | 0.989 | 0.734 | 0.786 | 0.946 |  | 193 | 31 | 70 | 6 | 0.862 | 0.921 | 0.970 | 0.693 | 0.720 | 0.952 | |
| Model 6*^f^* | 718 | 59 | 180 | 7 | 0.924 | 0.963 | 0.990 | 0.753 | 0.812 | 0.957 |  | 215 | 23 | 58 | 4 | 0.903 | 0.935 | 0.982 | 0.716 | 0.765 | 0.966 | |

*^a,b,c^*RP models are constructed based on molecular properties and FPFP_4 fingerprint; *^d,e,f^*Bayesian models are constructed based on molecular properties and LCFP_6 fingerprint; *^a,d^*The compound was categorized into inhibitor when mTOR inhibition value is less than 1 µM; *^b,e^*The compound was categorized into inhibitor when mTOR inhibition value is less than 5 µM; *^c,f^*The compound was categorized into the inhibitor when mTOR inhibition value is less than 10 µM.
