# Supplementary material for: Predicting mTOR Inhibitors with a Classifier Using Recursive Partitioning and Naïve Bayesian Approaches
Source: PLoS One. 2014 May 12;9(5):e95221. doi: 10.1371/journal.pone.0095221 (PMC4018356; doi:10.1371/journal.pone.0095221)
Supplement: Text S1 — Detailed information of of ACFs-NB algorithm. (DOC) [file pone.0095221.s008.doc]

**ACFs-NB algorithm**

For each compound, the ACFs were derived with the following steps:

(1) each heavy atom (non-hydrogen atom) was taken as an atom center for an ACF;

(2) atoms n-bonds (n ≥ 1) away from the center atom were taken, keeping the bonding topology inside the ACF. If n is 1, it is called as level one ACF (ACF1); if n is 2, it is called as level two ACF (ACF2); and so on.

Usually, ACFn+1 is larger than ACFn. Larger ACFs are structurally more specific and result in more accurate prediction, but lose universality. To find a balance point of the accuracy and universality, we generated ACF1-6 fragments from the data set using our in-house program.

Now, a compound is represented in an integer array, in which a component of the array is assigned with the number of occurrences for a corresponding ACF. In next step, we would calculate global inhibition and non-inhibition expectations for each ACF. Then, ACFs were used as descriptors to construct the naïve bayesian classification model.

Based upon Bayes' theorem, P(A|B) = P(B|A)P(A)/P(B),the probability of a compound inhibition (p) can be calculated as the following:


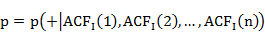


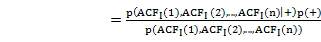
 (4)

where p(ACFI(1),ACFI(2),...,ACFI(n)|+) is the probability that a compound has a set of ACFs which are responsible for chemical inhibition. p(+) is the prior probability(see equation (11)), a probability derived from the training set, and p(ACF_I_(1),ACF_I_(2),...,ACF_I_(n)) is the marginal probability that given ACFs appear in the data set.

In a naïve Bayesian classifier, descriptors are independent to each other. Thus, the probability of a compound inhibition p(ACF_NI_(1),ACF_NI_(2),...,ACF_NI_(n)│+), and non-inhibition p(ACF_I_(1),ACF_I_(2),...,ACF_I_(n)│-), can be calculated as follows:


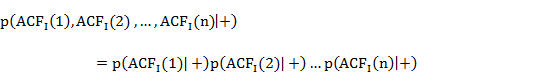


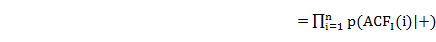
 (5)


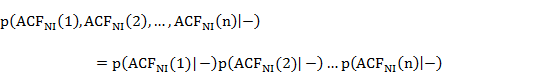


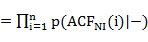
 (6)

Zero counts (ACFs only appear in inhibition class or *vice versa*) were treated by applying Laplacian correction and where (5) and (6)can be calculated as follows:


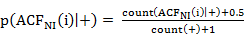
 (7)


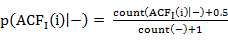
 (8)

The experimental data were divided into inhibition and non-inhibition classes. The non-inhibition probability q can be calculated in (9):


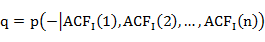


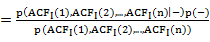
 (9)

Since p + q = 1, we have:


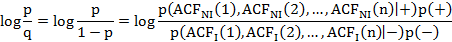


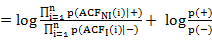
 (10)


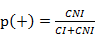
 (11)


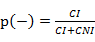
 (12)

Missing values (ACFs appear in query structure, but cannot be found in the knowledge base) were skipped. The program was developed in our lab and can be applied to classify the mTOR inhibitors and non-inhibitors. The program can be obtained by request.

**REFERENCES**

1. Yan X, Gu Q, Lu F, Li J, Xu J (2012) GSA: a GPU-accelerated structure similarity algorithm and its application in progressive virtual screening. Mol Divers 16: 759-769.

2. J X (1997) C-13 NMRspectral prediction by means of generalized atom center fragment method. Molecules 2: 114-128.

3. Sun H (2005) A naive bayes classifier for prediction of multidrug resistance reversal activity on the basis of atom typing. J Med Chem 48: 4031-4039.

4. Prathipati P, Ma NL, Keller TH (2008) Global Bayesian Models for the Prioritization of Antitubercular Agents. J Chem Inf Model 48: 2362-2370.
